# Feeding a Mixture of Choline Forms to Lactating Dams Improves the Development of the Immune System in Sprague-Dawley Rat Offspring

**DOI:** 10.3390/nu9060567

**Published:** 2017-06-02

**Authors:** Caroline Richard, Erin D. Lewis, Susan Goruk, Emily Wadge, Jonathan M. Curtis, René L. Jacobs, Catherine J. Field

**Affiliations:** 1 Department of Agricultural, Food and Nutritional Science, University of Alberta, Edmonton, AB T6G 2E1, Canada; Erin.Lewis@tufts.edu (E.D.L.); sgoruk@ualberta.ca (S.G.); wadge@ualberta.ca (E.W.); jcurtis1@ualberta.ca (J.M.C.); rjacobs@ualberta.ca (R.L.J.); cjfield@ualberta.ca (C.J.F.); 2 Jean Mayer United States Department of Agriculture, Human Nutrition Center on Aging, Tufts University, Boston, MA 02111, USA

**Keywords:** choline, phosphatidylcholine, glycerophosphocholine, immunology, spleen, lactation period, growth, Sprague-Dawley rat

## Abstract

Dietary choline is essential during lactation, but few studies have examined the implications of feeding a mixture of choline forms on immune function. This study investigates the impact of feeding lactating dams different mixtures of choline forms, similar to those in human diets, on the development and later immune function of suckled offspring. Sprague-Dawley lactating dams (*n* = 6/diet) were randomized to consume one of three diets, containing 1 g/kg choline: Control (100% free choline (FC)), Mixed Choline (MC: 50% phosphatidylcholine (PC), 25% FC, 25% glycerophosphocholine (GPC)), or High GPC (HGPC: 75% GPC, 12.5% PC, 12.5% FC). At weaning, female pups (*n* = 2/dam) were fed the Control diet until 10 weeks. At 3 weeks, MC and HGPC pups were heavier and their splenocytes had a higher proportion of helper T cells expressing CD25 and CD28 and produced less interferon gamma (IFN-γ) and tumor-necrosis factor-α (TNF-α) after Concanavalin A stimulation vs. Control pups (*p* < 0.05). At 10 weeks, MC and HGPC offspring had a lower proportion of macrophages and dendritic cells and produced less interleukin (IL)-1β but more IL-10 after lipopolysaccharide stimulation vs. Control pups (*p* < 0.05). In summary, feeding mixed choline diets during lactation improved T cell phenotype/function at the end of suckling and programmed a less inflammatory response later in life.

## 1. Introduction

Choline is an essential nutrient involved in lipid transport, methyl group donation, and the synthesis of acetylcholine and membrane phospholipids (reviewed in [1]). Although choline can be created through de novo synthesis, the amount of biosynthesis that occurs in the body cannot sufficiently meet the daily requirements for humans, especially during critical periods of rapid development such as pregnancy and lactation [2]. The dietary Adequate Intake (AI) values for choline were set at 425 mg/day for women and increase during pregnancy and lactation to 450 mg/day and 550 mg/day, respectively [3]. During pregnancy, choline is needed for the development of spinal cord structure and function in the fetus [4]. Substantial development also occurs during the suckling/lactation period for the brain [4], intestine/liver [5], and the immune system [6]. Using the Sprague-Dawley rat as a healthy rodent model, we have demonstrated that choline is required in the maternal diet during lactation for optimal immune function in the mother [7] as well as for the development of the immune system in their offspring [8]. This confirmed that an exogenous supply of choline during lactation is essential for infant and maternal immune function.

Choline exists in the diet in water-soluble forms (free choline (FC), phosphocholine, and glycerophosphocholine (GPC)) and lipid soluble forms (phosphatidylcholine (PC), lysophosphatidylcholine (lysoPC), and sphingomyelin (SM)) and as betaine, the choline metabolite that is formed upon the irreversible oxidation of choline [9]. Biochemically, each of these choline metabolites are absorbed [10] and metabolized differently and could contribute differently to the important roles of choline in the body. We have shown in our maternal cohort [11] that lactating women consumed different forms of choline; the most abundant forms being PC (46% of choline in the diet), FC (23%), and GPC (19%). For rodents, choline is typically provided as FC in standard chow diet (1 g/kg of diet) [12]. We have recently demonstrated that providing adequate amounts of choline as PC to lactating dams, compared to FC, improved the development of the immune system in suckled pups [13]. Although this suggests that the different forms of choline do not result in the same health benefits, it is not representative of human diet in which mixtures of choline forms are being consumed. To date, no study has examined the effect of feeding a combination of the major forms of choline metabolites in the maternal diet during lactation on the infant’s immune system development early in life.

The objectives of the current study were to determine the impact of different mixtures of choline forms in the maternal diet during lactation on (1) the development of the immune system in rat offspring at the end of the suckling period and (2) the potential programming effect on the offspring’s immune system later in life. We assessed experimental diets in which lactating dams were fed the recommended amount of total choline (1 g/kg, American Institute of Nutrition (AIN) recommendations [14]) provided as (1) a mixture of choline forms representative of human consumption (Mixed Choline diet (MC): 50% PC, 25% FC, 25% GPC), (2) a diet providing choline primarily as GPC (High GPC diet (HGPC): 75% GPC, 12.5% PC, 12.5% FC), or (3) a control (free choline diet: 100% FC). Providing a maternal diet containing 75% GPC was used to assess the specific impact of GPC on the immune system development, as it has not previously been investigated. GPC is a major form of choline in milk and although there is some interest in providing it in supplements, the HGPC diet is not representative of human consumption. We hypothesized that feeding a maternal diet containing a mixture of choline forms during lactation would have a beneficial effect on the development of the immune system in offspring compared to a standard rodent diet containing only free choline.

## 2. Materials and Methods

### 2.1. Animals and Diets

All animal care and experimental protocols were conducted in accordance with the Canadian Council on Animal Care and approved by the Committee of Animal Policy and Welfare of the University of Alberta, Edmonton, Alberta, Canada. The ethic approval code is AUP00000125_REN5. Female Sprague-Dawley rats (*n* = 18) were obtained from Charles River Laboratories (Montreal, QC, Canada) on day 14 of gestation and were individually housed in a temperature and humidity controlled environment with a 12/12-h reversed light cycle. Dams were fed standard rat chow (Lab diet 5001; PMI Nutrition International, Brentwood, MO, USA) throughout gestation, then randomized to one of three nutritionally complete experimental diets (Supplementary Table S1) 24–48 h prior to parturition: Control diet, Mixed Choline diet (MC), and High GPC diet (HGPC) (described below). At birth, the litters were culled to 10 pups per dam and diets were fed to dams ad libitum throughout lactation. Offspring were kept with their mothers for the duration of the suckling period (21 days). At 3 weeks, dams and pups (8/dam) were terminated and two pups from the same dam (1 male and 1 female) were pooled to represent a measure of the dam since the experimental unit in the study was the dam. In order to assess the potential programming effect, at the end of the suckling period, two pups from each dam (only females were kept) remained in the study and all consumed the Control diet, regardless of their previous suckling diet, for an additional seven weeks until adulthood was reached. At 10 weeks, the two pups from the same dam were terminated and measures were pooled to represent a measure for the dam, the experimental unit. This study design enabled us to specifically investigate the impact of feeding different forms of choline in the maternal diet during lactation (1) on the immune system development in offspring at the end of the suckling period and (2) the programming effect on immune development and function in offspring later in life.

The three experimental diets all contained 1 g of total choline/kg of diet, were isocaloric, isonitrogenous, and the macronutrient and micronutrient composition were identical differing only in the forms of choline provided (Supplementary Table S1): Control diet (FC: 1 g/kg of choline provided as 100% FC; *n* = 6), Mixed choline diet (MC: 1 g/kg of choline provided as 50% PC, 25% FC, 25% GPC; *n* = 6), High GPC (HGPC: 1 g/kg of choline provided as 75% GPC, 12.5% PC, 12.5% FC; *n* = 6). FC was added to the diets as choline bitartrate (a choline salt, Harlan Teklad, Frederick, MD, USA), PC as soy lecithin (MJS Biolynx, Brockville, ON, Canada), and GPC in a purified form (Santa Cruz Biotechnology, Dallas, TX, USA). As PC provided some lipids, the lipid content and composition of the experimental diets were adjusted to ensure that the diets contained similar fat content (% w/w) and fatty acid composition (Supplementary Table S2). The added fat mixture to the rodent diet was composed of flaxseed oil, sunflower oil, hydrogenated canola oil, olive oil, canola oil, vegetable oil, corn oil, a high arachidonic acid (AA) oil, and a high docosahexaenoic acid (DHA) oil (both AA and DHA oils were provided by DSM (Nutritional Products, Columbia, MD, USA) and all fatty acids were matched closely between diets. All three diets met the essential fatty acid requirements of the rodent [12] and had similar omega-6/omega-3 ratios and polyunsaturated fatty acids (PUFA)/saturated fatty acid (SFA) ratios. The basal composition of the three diets has been previously described [15]. Diets were prepared weekly and stored at 4 °C until fed; animals had free access to food throughout each 24 h period and feed cups were replaced every 2–3 days to prevent oxidation. Dietary intake and body weights were monitored regularly throughout the intervention.

### 2.2. Tissue Collection

At 3 weeks and 10 weeks, two offspring per dam were weighed and euthanized by CO_2_ asphyxiation in the morning hours. Spleens were collected aseptically, weighed, and immune cells were isolated for further processing (see Section 2.5). Offspring stomach contents and livers were collected aseptically, weighed, snap frozen in liquid nitrogen, and stored at −80 °C until analysis.

### 2.3. Choline Metabolite Analyses of Offspring Stomach Content and Splenocytes

Stomach contents of pups were analyzed to reflect the choline concentration in the pup’s diet at the end of the lactation period. Splenocytes and stomach contents were extracted using a modified Bligh and Dyer method that has been previously described in [16,17]. Extracts were quantified for all significant choline-containing metabolites and total choline content by hydrophilic interaction liquid chromatography (HILIC) liquid chromatography-tandem mass spectrometry (LC-MS/MS) using an Agilent 1200 series high performance liquid chromatography (HPLC) system (Agilent Technologies, Palo Alto, CA, USA) coupled to a 3200 QTRAP mass spectrometer (AB SCIEX, Concord, ON, Canada). The choline content in splenocytes was adjusted for protein content, which was measured using a commercial bicinchoninic acid (BCA) assay kit (Thermo Fisher Scientific, Edmonton, AB, Canada) according to manufacturer’s instructions.

### 2.4. Fatty Acid Analysis

The fatty acid composition of the three experimental diets was measured by gas chromatography. Briefly, total lipids from diets were extracted using Folch [18], saponified, and then methylated with hexane and BF_3_. The upper phase containing the lipids was dried under nitrogen, reconstituted in hexane, and the fatty acids were separated by gas chromatography.

### 2.5. Immune Cell Isolation

Isolation of immune cells from spleen has been previously described [19]. Briefly, single cell suspensions were obtained by disrupting tissue through a nylon mesh screen in sterile Krebs-Ringer 4-(2-hydroxyethyl)-1-piperazineethanesulfonic acid (HEPES) buffer with bovine serum albumin (5 g/L; Sigma-Aldrich Canada Ltd., Oakville, ON, Canada). Ammonium chloride lysis buffer (155 mM NH_4_Cl, 0.1 mM ethylene-diamine-tetra-acetic acid (EDTA), 10 mM KHCO_3_; Fisher Scientific, Edmonton, AB, Canada) was used to lyse erythrocytes. Cells were washed then re-suspended in complete culture medium (Roswell Park Memorial Institute (RPMI) 1640 media; Life Technologies, Burlington, ON, Canada), supplemented with 5% (*v/v*) heat-inactivated fetal calf serum, 25 mM HEPES, 2.5 mM 2-mercaptoethanol, and 1% antibiotic/antimycotic (pH 7.4; Fisher Scientific, see above). A haemocytometer was used to count live cells using trypan blue dye exclusion (Sigma-Aldrich, as above) and diluted to 1.25 × 10^6^ cells/mL.

### 2.6. Immune Cell Phenotype Analysis

Immune cell subsets present in freshly isolated splenocytes were identified by direct immunofluorescence assay, as previously described [20]. Briefly, immune cells (200,000) were incubated for 30 min at 4 °C with pre-labelled monoclonal antibodies applied in combination to quantify various immune cell phenotypes. The use of four-color flow cytometry allowed the identification of the following combinations of surface molecules in splenocytes: CD28/CD3/CD8/CD4, CD28/CD152/CD8/CD4, CD25/CD152/CD8/CD4, CD25/CD127/CD8/CD4, CD27/CD8/CD4, CD27/OX12/OX6/CD45ra, CD71/CD8/CD4, OX12/OX6/CD80, CD86/CD80/CD45ra, CD68/CD284/CD11b/c, OX62/CD25/OX6, CD161/OX62/CD3, IgG/IgM, IgA. All antibodies with the exception of IgG, IgM, and OX6 (BD Biosciences, Mississauga, ON, Canada) were purchased from Cedarlane Laboratories, (Burlington, ON, Canada). After incubation, cells were washed and fixed in paraformaldehyde (10 g/L; ThermoFisher, Edmonton, AB, Canada) in phosphate-buffered saline. All of the samples were acquired within 72 h of preparation by flow cytometry (FACSCalibur; Becton Dickinson, San Jose, CA, USA) according to the relative fluorescence intensity determined using Kaluza Software (Beckman Coulter, Mississauga, ON, Canada).

### 2.7. Ex Vivo Cytokine Production by Mitogen-Stimulated Splenocytes

The quantification of cytokines produced by mitogen-stimulated splenocytes has been previously described [21]. Briefly, cells (1.25 × 10^6^ cells/mL) were cultured in 3 mL RMPI-1640 medium (as above) for 48 h at 37 °C and 5% CO_2_ without mitogen (unstimulated) or with mitogen ConA (5 µg/mL; MP Biomedicals, Montreal, QC, Canada) or lipopolysaccharide (LPS, 100 µg/mL; Sigma-Aldrich, as above). ConA is a polyclonal T-cell stimulant and LPS activates the antigen-presenting cell population, including dendritic cells, macrophages, and B cells by binding to their Toll-like receptor-4 (CD284). After incubation for 48 h, cells were centrifuged for 10 min at 350 × g and supernatants were collected and stored at −80 °C until analyses. Concentrations of cytokines interleukin (IL)-1β, IL-2, IL-6, IL-10, tumor-necrosis factor-α (TNF-α), and interferon-γ (IFN-γ) were measured by commercial ELISA kits according to manufacturer’s instructions and as previously described [21]. The detection limits for all cytokines were 15.6–4000 pg/mL, except for IFN-γ in which the detection limit was 9.8–2500 pg/mL (R&D Systems, Minneapolis, MN, USA). Cytokine concentrations were quantified using a microplate reader (SpectraMax 190; Molecular Devices, Sunnyvale, CA, USA) and all measurements were conducted in duplicates, with coefficient of variation (CV) < 10%. The amount of IL-2 in the media after LPS stimulation was below detection levels. IL-1β was only measured in the supernatant of LPS stimulated cells.

### 2.8. Statistical Analyses

Data is reported as mean ± standard error of the mean (SEM) unless indicated otherwise. Based on previous studies using a similar model [8,13], it is estimated that five to six rats per diet group are required to assess significant changes in immune function (i.e., ex vivo cytokine production as the primary outcome). Data were analyzed using a one-way ANOVA in SAS (v9.4, Cary, NC, USA) with diet as the main effect. In cases where a significant main diet effect was found, post hoc analysis was performed using the DUNCAN adjustment to determine differences among diet groups. Variables not normally distributed were log-10 transformed prior to statistical analysis. Differences at *p* ≤ 0.05 (two-sided) were considered significant.

## 3. Results

### 3.1. Growth Parameters and Food Intake

There was no significant difference in dams’ body weight at the end of pregnancy (338 ± 7 g Control, 332 ± 12 g MC, and 339 ± 9 g HGPC, *p* = 0.498). There was also no difference in dams’ body weight at the end of the suckling period (302 ± 11g Control, 322 ± 9 g MC, and 318 ± 5 g HGPC, *p* = 0.284). Mean daily food intake of the dams in each diet group for the duration of the lactation period (21 days) was 47 ± 3 g/day, 46 ± 1 g/day, and 47 ± 1 g/day in the Control, MC, and HGPC group, respectively (*p* = 0.395). At the end of the suckling period, pups from dams fed MC or HGPC diets had higher body weight, spleen weight, and liver weight compared to pups from dams fed the Control diet (all *p* < 0.05, Table 1). Spleen weight of pups from dams fed MC or HGPC were also proportionally larger relative to their body weight compared to pups from dams fed Control diet. No significant differences in intestinal length and relative number of splenocytes (number of splenocytes/g spleen) were observed among diet groups at 3 weeks (Table 1). At 10 weeks of age, there were no significant differences in body weight (265.8 ± 7.2 g), spleen weight (0.80 ± 0.03 g), live weight (12.4 ± 0.5 g), intestinal length (116.4 ± 2.0 cm), and relative number of splenocytes (21.7 ± 3.0 10^6^/g spleen) among diet groups (*n* = 18). Mean daily food intake of the 10-week-old pups in each diet group was 32.5 ± 7 g/day, 31.9 ± 7 g/day, and 31.2 ± 8 g/day in the Control, MC, and HGPC group, respectively (*p* = 0.482).

### 3.2. Choline Metabolites in Pups’ Stomach Content

**Figure 1:**
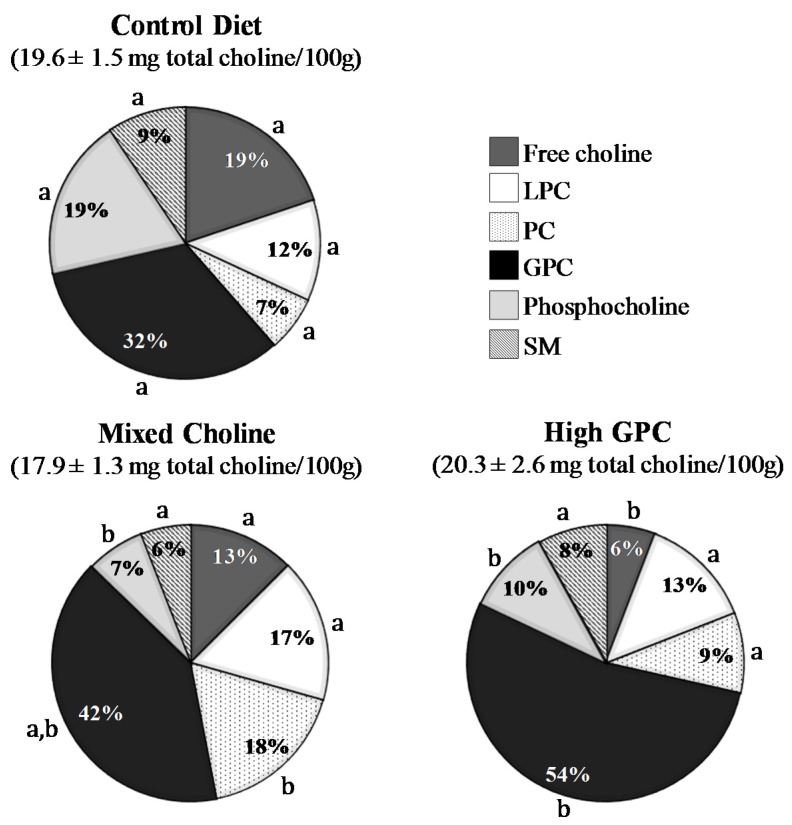
Contribution of choline-containing metabolites to total choline from 3-week-old pups’ stomach content. Values are presented as a percentage of contribution to total choline content (*n* = 6 per diet group). GPC, glycerophosphocholine; LPC, lysophosphatidylcholine; PC, phosphatidylcholine; SM, sphingomyelin; *p* value of the main effect of diet from the one-way ANOVA in SAS for total choline (*p* = 0.657), free choline (*p* = 0.003), LPC (*p* = 0.052), PC (*p* < 0.001), GPC (*p* = 0.022), phosphocholine (*p* =< 0.001), and SM (*p* = 0.216). Means that do not share a common superscript letter are significantly different (*p* < 0.05) according to post hoc analysis using the DUNCAN adjustment. Analysis performed on log-transformed values for LPC and PC.

Total choline concentration in pups’ stomach content (representative of pups’ diet at the end of the suckling period) was not significantly different among diet groups. Total choline concentration was 19.6 ± 1.5 mg/100 g, 17.9 ± 1.3 mg/100 g, and 20.3 ± 2.6 mg/100 g in pups’ stomach content from dams fed the Control, the MC, or the HGPC diets, respectively (Figure 1). The MC and HGPC diets had a lower proportion of phosphocholine compared to the Control diet in pups’ stomach content (all *p* < 0.05). The HGPC diet also resulted in a lower proportion of free choline when compared to the Control diet. There was a higher proportion of PC and GPC in pups’ stomach content from dam fed the MC and the HGPC diet, respectively (both *p* < 0.05, Figure 1).

### 3.3. Ex Vivo Cytokine Production by Stimulated Splenocytes

**Figure 2:**
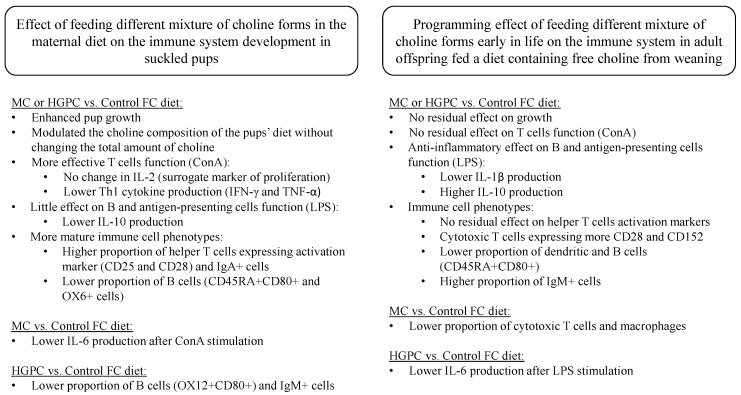
Summary of the effects of feeding different mixtures of choline forms in the maternal diet during the suckling period on the immune system development. CD, cluster of differentiation; ConA, Concanavalin A; Control, free choline; GPC, glycerophosphocholine; HGPC, high GPC; IL, interleukin; LPS, lipopolysaccharide; MC, mixed choline; TNF-α, tumor necrosis factor alpha.

Ex vivo cytokine production by ConA- and LPS-stimulated splenocytes isolated from 3-week-old pups is presented in Table 2. Following stimulation with a T cell mitogen ConA, splenocytes from pups from dams fed the MC and the HGPC diets produced significantly less IFN-γ and TNF-α compared to pups from Control-fed dams (both *p* < 0.05, Figure 2). Feeding a MC diet also led to a lower production of IL-6 by ConA-stimulated splenocytes in 3-week-old pups compared to the Control diet (*p* < 0.05, Figure 2). At 3 weeks, there was no change in the Ex vivo production of IL-2 and IL-10 by splenocytes after ConA stimulation among diet groups (Table 2). Following stimulation with LPS (bacterial challenge), splenocytes from 3-week-old pups from dams fed the MC and the HGPC diets produced significantly less IL-10 compared to pups from Control-fed dams (*p* < 0.05, Table 2). No change was observed in the production of IL-1β, IL-6, and TNF-α by splenocytes among diet groups after LPS stimulation in 3-week-old pups (Table 2). For both ConA- and LPS-stimulated splenocytes, no differences were observed in cytokine production between the MC and the HGPC diets. There was no significant difference in the concentration of total choline adjusted for protein content (*n* = 18, 4.1 ± 0.2 µg/mg of protein) in splenocytes among diet groups in 3-week-old pups. There was also no significant change in the proportion of choline coming from the different forms of choline (i.e., FC, PC, Lyso-PC, GPC, phosphocholine, and sphingomyelin) in splenocytes among diet groups.

Ex vivo cytokine production by ConA- and LPS-stimulated splenocytes isolated from 10-week-old offspring are presented in Table 3. There was no significant change among diet groups in the ex vivo production of IL-2, IL-6, IL-10, IFN-γ, and TNF-α by splenocytes after ConA stimulation at 10 weeks. After LPS stimulation, offspring that received the MC and the HGPC diets at suckling produced less IL-1β but more IL-10 compared to the Control diet (all *p* < 0.05). Offspring from dams fed the HGPC diet also produced less IL-6 in response to LPS. There were no differences in cytokine production between the MC and the HGPC diets in 10-week-old offspring. There was no significant difference in the concentration of total choline adjusted for protein content (*n* = 18, 4.9 ± 0.3 µg/mg of protein) in splenocytes among diet groups. There was also no significant change in the proportion of choline coming from the different forms of choline (i.e., FC, PC, Lyso-PC, GPC, phosphocholine, and sphingomyelin) in splenocytes among diet groups.

### 3.4. Splenocyte Immune Cell Phenotypes

At 3 weeks of age, there was no change in the proportion of total T cells (CD3+) and helper T cells (% of CD3+ cells that also express CD4) and cytotoxic/suppressor T cells (% of CD3+ cells that also express CD8) among groups (Table 4). However, pups from dams fed the MC and the HGPC diets had a higher proportion of helper T cells expressing CD25 (IL-2 receptor) and CD28 (co-stimulatory molecule) and IgA+ cells, and a lower proportion of IgG+, OX6+ (MHC Class II), and CD45RA+CD80+ cells compared to pups from dams fed the Control diet (all *p* < 0.05). Pups from dams fed the HGPC diet also had a lower proportion of IgM+ cells, OX6+CD80+ cells, and OX12+CD80+ cells compared to the Control diet (all *p* < 0.05, Table 4). No differences were observed in immune cells phenotypes between the MC and the HGPC diets.

At 10 weeks of age, there was no change in the proportion of total T cells and helper T cells among groups (Table 5). Offspring from dams that were fed a mixture of choline forms had a lower proportion of cytotoxic T cells expressing CD28, but a higher proportion of cytotoxic T cells expressing CD152 (cytotoxic T-lymphocyte-associated protein 4) vs. the Control diet. Offspring that received the MC diet also had a lower proportion of cytotoxic T cells compared to the Control diet. Both MC and HGPC diets also led to a higher proportion of IgM+ cells and a lower proportion of dendritic cells (OX62+OX6+) and CD45RA+CD80+ cells vs. FC diet (all *p* < 0.05). Compared to the Control diet, offspring that received the MC diet had a lower proportion of macrophages (CD68+) while those that received the HGPC diet had a lower proportion of OX12+CD80+ and IgA+ cells (all *p* < 0.05). No differences were observed in immune cell phenotypes between the MC and the HGPC diets.

## 4. Discussion

We investigated for the first time the impact of feeding a maternal diet containing different forms of choline during the lactation period on the development of the immune system in suckled pups and the potential programming effect on the immune system later in life. In the current study, we used data from our maternal pregnant and lactating women cohort [11] to design a rodent diet containing a distribution of choline forms representative of human consumption, the MC diet (50% PC, 25% FC, and 25% GPC) and we also designed an experimental diet high in GPC (75% GPC, 12.5% FC, and 12.5% PC), a form of choline that is high in human, rodent, and cow’s milk [22]. We demonstrated that feeding a MC diet or a HGPC diet to lactating dams enhances the growth of their offspring at the end of the suckling period. Kawamura et al. [23] have shown in young adults when compared to a placebo, that providing a bolus of GPC increased plasma levels of the growth hormone which is known to stimulate growth, cell reproduction, and tissue regeneration. This suggests that GPC may be a key molecule in promoting pups’ growth, since both maternal diets increased the proportion of GPC in breast milk and, as previously reported, had no effect on pups’ growth by feeding a maternal diet containing only PC [13]. Birth weight was not recorded, which is a limitation of the current study, although based on the timing of the dams’ diet (24–48 h prior to parturition) and the fact that there was no difference in dams’ body weight at the end of pregnancy, it is unlikely that there was difference in pups’ body weight at birth. As summarized in Figure 2, we showed that feeding a MC diet and a HGPC diet to lactating dams led to similar beneficial changes in immune development and function in pups at the end of the suckling period (i.e., 3-week-old pups) and influenced the immune response in adulthood (i.e., 10-week-old offspring).

We showed that feeding different forms of choline in the maternal diet when providing the same amount of total choline had no impact on the total choline concentration in pups’ stomach content. This is consistent with our previous study in which feeding a maternal diet containing the same amount of total choline provided as either PC or as FC did not significantly affect the total choline concentration in pups’ stomach content [13]. However, changing the forms of choline in the maternal diet significantly altered the distribution of choline forms in pup’s stomach content. We showed that feeding a MC diet mainly composed of PC (50% of the diet) increased the proportion of PC (by 11%) with a reciprocal reduction in the proportion of phosphocholine (by 12%). We also showed that feeding a HGPC diet mainly composed of GPC (75% of the diet) led to a higher proportion of GPC (by 21%) that was counterbalanced by a reduction in the proportion of FC (by 14%) and phosphocholine (by 9%). Altogether, our results demonstrate that feeding different forms of choline in the maternal diet during lactation modulated the pup’s diet, which likely contributed to the immune differences observed among groups. However, we only measured the choline composition in pups’ stomach content and, therefore, it is possible that some of the immune benefits observed might have been attributable to other changes that were not measured. We had previously demonstrated that feeding a maternal diet containing 100% PC led to a higher proportion of PC in the offspring’s splenocytes [13]. In this study, providing a mixture of choline forms (as opposed to 100% of a single form) was not enough to induce a significant difference in the splenocytes choline composition while still having important effects on immune function.

### 4.1. Impact of Choline Forms in the Maternal Diet on the Development of the Immune System Early in Life

Compared to adults, neonatal T cells require more co-stimulation in order to mount an efficient T helper 1 (Th1) response (IL-2, IFN-γ, and TNF-α), both in vivo and ex vivo [24]. Maturation during infancy is associated with not only an increased proportion of T cells expressing activation markers, but with a higher proliferative response (reviewed in [25,26]). Although the proportion of total T cells did not differ among groups, there were a higher proportion of helper T cells expressing activation markers such as the IL-2 receptor (CD4+CD25+) and the co-stimulatory molecule (CD4+CD28+) in pups from dams fed both the MC and the HGPC diets at the end of the suckling period. We have shown that the forms of choline in the maternal diet did not affect IL-2 production following stimulation by a T cell mitogen (ConA), which is a pleiotropic Th1 cytokine involved in many critical immune responses but most importantly in T cell growth and proliferation (reviewed in [27]). Nonetheless, pups from the dams fed MC and HGPC diets produced less key immunomodulatory Th1 cytokines including IFN-γ (by 44% to 66% less) and TNF-α (by 50% less) in response to ConA. Therefore, it appears that pups fed a mixture of choline forms during suckling had an overall more efficient/mature immune response to challenge in that they did not have to produce as much Th1 cytokines in order to maintain IL-2 production (a surrogate marker of proliferation). This could be explained, at least partly, by the fact that pups fed a mixture of choline forms had a higher proportion of helper T cells expressing activation markers (i.e., CD25 and CD28) required for T cell activation and proliferation [28].

Similar to humans [26], during the early suckling period in rodents, there is a predominance of B cells (CD45RA+ and OX12+ cells), while the proportion of T cells and NK cells in spleen are lower than in adults [25]. At birth, the immune system is also characterized by a predominant Th2 cytokine response (IL-4, IL-5, IL-10, and IL-13) that promotes a humoral response by B cells involving the production of antibodies (i.e., immunoglobulins, Ig) (reviewed in [29,30]). Although there was no change in the proportion of macrophages and dendritic cells, pups from dams fed the MC and the HGPC diets had a lower proportion of MHC class II + cells (OX6 expressed on antigen-presenting cells (APCs) including B cells), B cells expressing CD80 (CD45RA+CD80+ and OX12+CD80+), and IgG+ and IgM+ B cells, while having a higher proportion of IgA+ B cells. Similar to humans, IgM-producing cells in rats are the first to appear while IgA-secreting cells are scarce during suckling and maturation during infancy is associated with a shift from IgM- to IgA-producing cells [25,26]. This overall more mature lymphocyte phenotypic distribution may be partly responsible for the lower Th2 response observed in pups from dams that received a mixture of choline forms. Indeed, pups from dams fed the MC diet produced 60% less IL-6 after ConA stimulation (did not reach significance with the HGPC diet). IL-6 is typically considered a Th2 cytokine having a dual effect on the Th1/Th2 balance by promoting Th2 differentiation and also inhibiting Th1 polarization [31]. IL-10 is another Th2 cytokine known to inhibit Th1 cell proliferation and IL-2 secretion [32,33]. In response to a bacterial challenge (LPS), we showed that pups that received the MC and the HGPC diets had a lower production of IL-10 (by 45% on average). Therefore, it is also possible that the lower production of Th2 cytokines (IL-6 and IL-10) may have contributed to maintaining Th1 cell proliferation (i.e., IL-2 production), while producing less immunomodulatory Th1 cytokines (i.e., IFN-γ and TNF-α).

### 4.2. Programming Effect of Feeding a Mixture of Choline Forms During Suckling on the Immune System Later in Life

Feeding a mixture of choline forms during the suckling period had a programming effect on the ability of B cells/APCs to respond after a bacterial challenge (LPS) in adult offspring that was not observed at 3 weeks of age. Indeed, at 10 weeks of age, offspring that received the MC and the HGPC diets during suckling produced 30% less IL-1β and about 70% and 130% more IL-10 after LPS stimulation, respectively. Feeding a HGPC diet at suckling also resulted in a lower production of IL-6 (35% less) after LPS stimulation, while not reaching statistical significance with the MC diet, suggesting that GPC might have a more potent anti-inflammatory effect than PC. Although the molecular mechanisms by which GPC exerts a programming anti-inflammatory effect remain to be elucidated, Tokes et al. [34] reported a protective effect of GPC against intestinal superoxide production after an ischaemia-reperfusion injury, thereby providing indirect evidence of the potential anti-inflammatory effect of GPC. The lower production of IL-1β and IL-6 could partly be explained by the lower proportion of total macrophages (CD68+ cells) and dendritic cells (OX62+OX6+ cells) observed in adult offspring that received a mixture of choline forms, since APCs are major producers of pro-inflammatory cytokines including IL-1β and IL-6 [35,36]. However, this pattern of cytokine production is more consistent with an overall anti-inflammatory effect, since IL-10 is also produced by activated macrophages and B cells [35] and feeding a mixture of choline forms (both MC and HGPC diets) during the suckling period led to an increased production of IL-10 along with a lower proportion of B cells (CD45RA+CD80+ and OX12+CD80+). IL-10 functions as an anti-inflammatory cytokine by suppressing macrophage activation and production of many pro-inflammatory cytokines (IL-1β, IL-6, and TNF-α) [37], whereas IL-1β is known to promote helper T cell differentiation and expansion [38]. Despite the lower IL-1β production, our results should not be interpreted as impaired immune function, since there was no change in the ability of T cells to produce IL-2 upon challenge (a surrogate marker of proliferation). Moreover, adult offspring that received the MC or HGPC diets at suckling had a higher proportion of IgM+ cells, which would be expected to be beneficial against microbial infection [39].

Although most of the effect of feeding a mixture of choline forms in suckled pups was observed on T cell function (i.e., after ConA stimulation), we found no programming effect on the ability of T cells to respond to challenge later in life. Indeed, offspring that received a mixture of choline forms at suckling no longer had a higher proportion of helper T cells expressing the IL-2 receptor (CD25) and the co-stimulatory molecule (CD28). Moreover, they had a lower proportion of cytotoxic T cells expressing CD28 along with a higher proportion of cytotoxic T cells expressing the cytotoxic T lymphocyte associated protein 4 (CD152). While the co-stimulatory molecule CD28 is essential for T cell activation, CD152 has an opposite effect by downregulating immune cell activation [28]. This could explain, at least partly, the modest programming effect of feeding a mixture of choline forms at suckling on T cells function later in life.

Finally, our results suggest that the forms of choline in the maternal diet during lactation, but also in infant formula for non-breast fed infants, need to be considered. Several differences exist regarding the forms of choline between human milk and both soy and cow-based infant formulas [22,40]. Although, human milk is known to contain more phosphocholine, it also contains 30% to 80% less free choline than infant formulas [22,40]. This is attributable to the fact that choline is mainly added to infant formulas as choline salt (choline bitartrate or choline chloride). Our study provides new immunological evidence that, despite the total amount of choline, the forms of choline in an infant’s diet early in life is also important. However, future studies are required to determine the ideal proportion of the different choline forms in infant formula for optimal immune function.

## 5. Conclusions

In summary, we demonstrated that the choline composition in the maternal diet influences immune development and function in the offspring. Feeding dams MC or HGPC diets, compared to Control diet providing the sole source of choline, resulted in a higher proportion of PC or GPC and less free choline in the pups’ stomach content. In suckled offspring, feeding a mixture of choline forms (both a MC or a HGPC diet) led to an overall more mature lymphocyte phenotype with more helper T cells expressing activation markers and less B cells, which resulted in a beneficial effect on T cell function in that pups fed a mixture of choline forms did not have to produce as much cytokines in order to maintain a normal proliferative response. In female adult offspring, providing a mixture of choline forms during the suckling period had an overall anti-inflammatory programming effect on B cells/antigen presenting cells function with lower pro-inflammatory and higher anti-inflammatory cytokine production along with a lower proportion of macrophages and dendritic cells. Our results highlight the importance of considering the different forms of choline in rodent diet and infant formula for optimal immune function.

## Figures and Tables

**Table 1:** Growth parameters at the end of the suckling period of 3-week-old pups from lactating dams fed the Control, the MC or the HGPC diet.

Variable	Control Diet (*n* = 6)	MC Diet (*n* = 6)	HGPC Diet (*n* = 6)	*p* Value
BW (g) ^1^	59.8 ± 3.4 ^a^	74.4 ± 2.8 ^b^	71.5 ± 2.5 ^b^	0.007
Spleen weight (g)	0.29 ± 0.02 ^a^	0.49 ± 0.04 ^b^	0.45 ± 0.02 ^b^	0.001
Spleen weight/BW (%)	0.47 ± 0.02 ^a^	0.65 ± 0.03 ^b^	0.63 ± 0.03 ^b^	0.002
Liver weight (g)	2.48 ± 0.15 ^a^	3.35 ± 0.15 ^b^	3.17 ± 0.16 ^b^	0.003
Liver weight/BW (%)	4.17 ± 0.18	4.50 ± 0.06	4.42 ± 0.11	0.205
Gut length (cm) ^1^	71.0 ± 1.4	77.3 ± 2.5	82.0 ± 5.6	0.132
Splenocytes (10^6^/g spleen)	14.5 ± 0.9	16.0 ± 1.8	14.7 ± 0.4	0.777

Values are presented as mean ± SEM; BW, body weight; Control, free choline; HGPC, high glycerophosphocholine; MC, mixed choline; *p* value of the main effect of diet from the one-way ANOVA in SAS. Means within a row that do not share a common superscript letter are significantly different (*p* < 0.05) according to post hoc analysis using the DUNCAN adjustment. ^1^ Analysis performed on log-transformed values.

**Table 2:** Ex vivo cytokine production by mitogen-stimulated splenocytes in 3-week-old pups from lactating dams fed the Control, the MC, or the HGPC diet.

Cytokine Concentration (pg/mL)	Control Diet (*n* = 6)	MC Diet (*n* = 6)	HGPC Diet (*n* = 6)	*p* Value
**ConA**				
IL-2 ^1^	2559 ± 323	2928 ± 318	2804 ± 486	0.383
IL-6	57 ± 9 ^a^	22 ± 7 ^b^	47 ± 8 ^a,b^	0.022
IL-10	72 ± 11	52 ± 9	82 ± 14	0.212
IFN-γ ^1^	71 ± 6 ^a^	24 ± 5 ^b^	40 ± 9 ^b^	0.010
TNF-α ^1^	132 ± 27 ^a^	65 ± 12 ^b^	65 ± 8 ^b^	0.022
**LPS**				
IL-1β ^1^	188 ± 14	238 ± 28	216 ± 12	0.303
IL-6	473 ± 28	536 ± 39	591 ± 53	0.147
IL-10	187 ± 22 ^a^	111 ± 20 ^b^	99 ± 13 ^b^	0.009
TNF-α	1176 ± 80	1297 ± 153	1203 ± 225	0.841

Values are presented as mean ± SEM; Control, free choline; HGPC, high glycerophosphocholine; IFN-γ, interferon gamma; IL, interleukin; MC, mixed choline; TNF-α, tumor necrosis factor alpha; *p* value of the main effect of diet from the one-way ANOVA in SAS. Means within a row that do not share a common superscript letter are significantly different (*p* < 0.05) according to post hoc analysis using the DUNCAN adjustment. ^1^ Analysis performed on log-transformed values.

**Table 3:** Ex vivo cytokine production by mitogen-stimulated splenocytes in 10-week adult offspring that were fed the FC, the MC or the HGPC diet during the suckling period.

Cytokine Concentration (pg/mL)	Control Diet (*n* = 6)	MC Diet (*n* = 6)	HGPC Diet (*n* = 6)	*p* Value
**ConA**				
IL-2	3202 ± 265	3843 ± 333	3401 ± 206	0.267
IL-6 ^1^	310 ± 47	468 ± 43	420 ± 59	0.086
IL-10 ^1^	150 ± 21	182 ± 31	263 ± 47	0.088
IFN-γ	504 ± 36	597 ± 90	607 ± 52	0.472
TNF-α ^1^	152 ± 14	166 ± 16	185 ± 30	0.691
**LPS**				
IL-1β ^1^	157 ± 13 ^a^	113 ± 11 ^b^	111 ± 9 ^b^	0.024
IL-6 ^1^	924 ± 88 ^a^	703 ± 40 ^a,b^	596 ± 85 ^b^	0.029
IL-10 ^1^	319 ± 35 ^a^	551 ± 65 ^b^	749 ± 99 ^b^	0.001
TNF-α	276 ± 41	295 ± 40	265 ± 41	0.868

Values are presented as mean ± SEM; Control, free choline; HGPC, high glycerophosphocholine; IFN-γ, interferon gamma; IL, interleukin; MC, mixed choline; TNF-α, tumor necrosis factor alpha; *p* value of the main effect of diet from the one-way ANOVA in SAS. Means within a row that do not share a common superscript letter are significantly different (*p* < 0.05) according to post hoc analysis using the DUNCAN adjustment. ^1^ Analysis performed on log-transformed values.

**Table 4:** Splenocyte immune cell phenotypes in 3-week-old pups from lactating dams fed the Control, the MC, or the HGPC diet.

Cell Phenotype	Control Diet (*n* = 6)	MC Diet (*n* = 6)	HGPC Diet (*n* = 6)	*p* Value
**% of total lymphocytes**				
Total CD3+ (T cells)	16.2 ± 0.8	18.5 ± 0.7	17.8 ± 0.7	0.373
**% of CD3+ cells**				
CD4+ (Helper T cells)	45.5 ± 1.0	42.5 ± 1.8	41.2 ± 1.6	0.189
CD8+ (Cytotoxic T cells)	40.7 ± 1.9	37.7 ± 1.1	39.3 ± 1.4	0.373
**% of CD4+ T cells**				
CD25+	8.0 ± 1.3 *^a^*	12.2 ± 0.7 *^b^*	11.5 ± 0.9 *^b^*	0.020
CD28+	82.1 ± 1.9 *^a^*	94.2 ± 1.1 *^b^*	94.7 ± 2.3 *^b^*	<0.001
**% of CD8+ T cells**				
CD25+	5.5 ± 1.0	5.0 ± 0.4	5.0 ± 0.4	0.816
CD28+	74.4 ± 2.8	74.6 ± 2.2	71.0 ± 1.1	0.438
**% of total lymphocytes**				
Total CD68+ (Macrophages)	9.6 ± 0.8	9.6 ± 0.4	8.9 ± 0.2	0.501
CD68+CD284+	3.3 ± 0.4	2.5 ± 0.1	2.1 ± 0.1	0.214
OX62+OX6+ (Dendritic cells)	3.6 ± 0.2	3.1 ± 0.5	2.6 ± 0.2	0.072
CD3-CD161+ (Natural Killer cells)	4.3 ± 0.4	3.6 ± 0.2	3.3 ± 0.2	0.802
Total CD45RA+ ^1^	36.2 ± 2.5	30.4 ± 1.4	31.3 ± 1.4	0.100
CD45RA+CD80+	4.8 ± 0.3 ^a^	3.9 ± 0.4 ^b^	3.3 ± 0.3 ^b^	0.013
Total OX6+ (MHC class II+)	40.8 ± 2.0 ^a^	35.2 ± 1.6 ^b^	34.9 ± 1.5 ^b^	0.046
Total OX12+ (B cells)	35.5 ± 1.6	31.5 ± 1.9	32.7 ± 1.7	0.267
OX12+CD80+	6.3 ± 0.4 ^a^	5.7 ± 0.5 ^a,b^	3.8 ± 0.4 ^b^	0.003
IgG+ ^1^	8.6 ± 0.7 ^a^	4.8 ± 0.7 ^b^	5.4 ± 0.6 ^b^	0.003
IgM+ ^1^	48.0 ± 2.2 ^a^	41.6 ± 2.1 ^a,b^	40.1 ± 1.8 ^b^	0.043
IgA+ ^1^	5.9 ± 0.5 ^a^	18.4 ± 1.6 ^b^	22.4 ± 1.8 ^b^	<0.001

Values are presented as mean ± SEM; Values are a proportion of the total gated cells as determined by immunofluorescence. CD, cluster of differentiation; Control, free choline; GPC, glycerophosphocholine; HGPC, high GPC; MC, mixed choline; *p* value of the main effect of diet from the one-way ANOVA in SAS. Means within a row that do not share a common superscript letter are significantly different (*p* < 0.05) according to post hoc analysis using the DUNCAN adjustment. No significant differences were observed among diet groups (*n* = 18; mean ± SEM) for total cells expressing CD25 (3.2 ± 0.3), CD127 (1.1 ± 0.2), CD152 (2.0 ± 0.3), CD71 (18.1 ± 1.7), CD11bc (8.6 ± 0.4), and CD284 (30.6 ± 0.7) or CD4+CD27+ (7.5 ± 0.5), CD8+CD27+ (7.1 ± 0.4), CD68+CD11bc+ (6.7 ± 0.4), CD4+CD152+ (0.4 ± 0.2), and CD8+CD152+ (0.4 ± 0.1) cells. ^1^ Analysis performed on log-transformed values.

**Table 5:** Splenocyte immune cell phenotypes in 10-week-old adult offspring that were fed the FC, the MC, or the HGPC diet during the suckling period.

Cell Phenotype	Control Diet (*n* = 6)	MC Diet (*n* = 6)	HGPC Diet (*n* = 6)	*p* Value
**% of total lymphocytes**				
Total CD3+ (T cells)	35.3 ± 1.5	37.1 ± 1.6	38.3 ± 1.9	0.649
**% of CD3+ cells**				
CD4+ (Helper T cells)	51.7 ± 1.2	54.9 ± 2.1	48.6 ± 2.4	0.117
CD8+ (Cytotoxic T cells)	43.7 ± 1.1 ^a^	38.1 ± 1.7 ^b^	39.4 ± 1.5 ^a,b^	0.039
**% of CD4+ T cells**				
CD25+	14.1 ± 1.2	15.7 ± 0.3	16.6 ± 1.3	0.252
CD28+	91.6 ± 1.0	87.9 ± 1.0	89.3 ± 1.1	0.073
CD152+	4.3 ± 0.3	5.7 ± 0.7	6.0 ± 0.9	0.159
**% of CD8+ T cells**				
CD25+	8.7 ± 0.4	10.8 ± 1.0	11.0 ± 0.9	0.127
CD28+	77.9 ± 1.9 ^a^	68.3 ± 2.2 ^b^	71.3 ± 2.2 ^b^	0.016
CD152+	3.7 ± 0.3 ^a^	6.8 ± 1.1 ^b^	6.5 ± 0.9 ^b^	0.037
**% of total lymphocytes**				
Total CD68+ (Macrophages)	13.5 ± 0.9 ^a^	10.3 ± 0.3 ^b^	11.7 ± 0.5 ^a,b^	0.001
CD68+CD284+	8.9 ± 0.4	8.0 ± 0.2	8.5 ± 0.3	0.138
OX62+OX6+ (Dendritic cells)	7.3 ± 0.4 ^a^	5.7 ± 0.5 ^b^	5.8 ± 0.6 ^b^	0.018
CD3-CD161+ (NK cells)	8.0 ± 0.7	6.8 ± 0.3	6.5 ± 0.3	0.071
Total CD45RA+	44.9 ± 1.2	44.8 ± 0.8	43.4 ± 1.4	0.583
CD45RA+CD80+	4.4 ± 0.3 ^a^	2.9 ± 0.1 ^b^	3.2 ± 0.3 ^b^	0.001
Total OX6+ (MHC class II+) ^1^	49.8 ± 2.6	49.1 ± 0.9	48.3 ± 1.3	0.868
Total OX12+ (B cells)	42.1 ± 1.9	42.9 ± 1.9	40.8 ± 2.2	0.767
OX12+CD80+	8.6 ± 0.2 ^a^	7.4 ± 0.5 ^a,b^	6.7 ± 0.5 ^b^	0.016
IgG+	10.6 ± 0.4	11.6 ± 0.4	11.4 ± 0.4	0.196
IgM+ ^1^	52.6 ± 1.5 ^a^	60.5 ± 0.8 ^b^	59.2 ± 0.9 ^b^	<0.001
IgA+ ^1^	10.3 ± 0.4 ^a^	9.2 ± 0.2 ^a,b^	8.6 ± 0.5 ^b^	0.016

Values are presented as mean ± SEM; Values are a proportion of the total gated cells as determined by immunofluorescence. CD, cluster of differentiation; Control, free choline; GPC, glycerophosphocholine; HGPC, high GPC; MC, mixed choline; *p* value of the main effect of diet from the one-way ANOVA in SAS. Means within a row that do not share a common superscript letter are significantly different (*p* < 0.05) according to post hoc analysis using the DUNCAN adjustment. No significant differences were observed among diet groups (*n* = 18; mean ± SEM) for total cells expressing CD127 (2.5 ± 0.4), CD71 (23.5 ± 0.6), CD11bc (10.9 ± 0.6), and CD284 (31.2 ± 1.0) or CD4+CD27+ (17.4 ± 1.3), CD8+CD27+ (16.2 ± 0.7), and CD68+CD11bc+ (3.7 ± 0.2) cells. ^1^ Analysis performed on log-transformed values.

## Supplementary Materials

The following are available online at www.mdpi.com/2072-6643/9/6/567/s1, Table S1: Composition of experimental diets fed to lactating dams; Table S2: Fatty acid composition of experimental diets fed to lactating dams.
