# Supplementary material for: Feeding a Mixture of Choline Forms to Lactating Dams Improves the Development of the Immune System in Sprague-Dawley Rat Offspring
Source: Nutrients. 2017 Jun 2;9(6):567. doi: 10.3390/nu9060567 (PMC5490546; doi:10.3390/nu9060567)
Supplement: Supplementary file 1 [file nutrients-09-00567-s001.pdf]

Table S1. Composition of experimental diets fed to lactating dams [1]

Component (g/kg diet)	Control Diet	MC Diet	HGPC Diet
Casein	270	270	270
Starch	240	240	240
Sucrose	126	126	126
Vitamin mix (AIN-93-Vx) [2]	19	19	19
Mineral mix [3]	50	50	50
Calcium phosphate dibasic	3.4	3.4	3.4
Inositol	6.3	6.3	6.3
Cellulose	80.0	80.0	80.0
L-cysteine	1.8	1.8	1.8
**Fat mixture**			
Canola oil	40	31	39
Olive oil	13	13.8	8.5
Vegetable oil	14	14	14.3
Corn oil	73	65	71.9
Sunflower oil	2	5	2.8
Flax seed oil	2	2	2
Hydrogenated Canola oil	56	57.5	56
DHAsco	1.5	1.5	1.5
ARAsco	1.5	1.5	1.5
**Total fat**	200	200	200
**Choline mixture (providing 1 g of total choline/kg diet)**			
Soy lecithin (PC)	0	13.1	3.2
Choline bitartrate	2.1	0.6	0.3
Glycerophosphocholine	0.0	0.6	1.9

[1] All ingredients were purchased from Harlan Teklad (Indianapolis, IN, USA), with the exception of the dietary oils that were all purchased from Safeway (Edmonton, AB, Canada) and hydrogenated canola oil that was donated by Richardson Oilseed Limited (Lethbridge, AB, Canada) and ARAsco (oil rich in arachidonic acid) and DHAsco (oil rich in docosahexaenoic acid) that were donated by DSM (Nutritional Products, Columbia, Maryland, US). FC, free choline; HGPC, high glycerophosphocholine; MC, mixed choline;

[2] AIN-93-VX Vitamin mix [1]

[3] Bernhart-Tomarelli salt mixture [2]

Table S2. Fatty acid composition of experimental diets fed to lactating dams[1]

Fatty acid	Control Diet	MC Diet	HGPC Diet
	g/100 g of total fatty acids		
C16:0	8.5	8.5	8.0
C16:1 n9	0.2	0.2	0.2
C18:0	24.5	26.3	24.8
C18:1 n9	31.8	31.4	32.4
C18:2 n6	29.3	28.3	28.9
C20:0	1.0	0.9	0.9
C18:3 n3 (ALA)	2.8	3.0	3.3
C20:3 n6	0.3	0.3	0.3
C20:4 n6 (AA)	0.3	0.3	0.3
C22:6 n3 (DHA)	0.3	0.3	0.3
Other fatty acids[2]	1.9	1.8	2.1
Total SFA	33.1	34.9	32.9
Total PUFA	33.2	31.9	32.7
Total n-6	29.5	28.6	29.2
Total n-3	3.6	3.3	3.6
Total MUFA	31.8	31.4	32.4
n-6/n-3	8.1	8.8	8.2
PUFA/SFA	1.0	0.9	1.0

[1] Analysis of the fatty acid composition of the three experimental diets collected weekly and pooled; AA, arachidonic acid; ALA, α-linolenic acid; DHA, docosahexaenoic acid; FC, free choline; HGPC, high glycerophosphocholine; MC, mixed choline; MUFA, monounsaturated fatty acids; n, omega; PUFA, polyunsaturated fatty acids; SFA, saturated fatty acids;

[2] other fatty acids refer to fatty acids that contributed for less than 0.2% in the diet which included trace of 10:0, 12:0, 14:0, 15:0, 18:1c11, 20:2n-6, 20:5n-3, 22:0, 22:4n-6, and 22:5n-3.

References

1. Reeves, P.G.; Nielsen, F.H.; Fahey, G.C., Jr. AIN-93 purified diets for laboratory rodents: Final report of the american institute of nutrition ad hoc writing committee on the reformulation of the AIN-76A rodent diet. *J. Nutr.* **1993**, *123*, 1939–1951.
2. Bernhart, F.W.; Tomarelli, R.M. A salt mixture supplying the national research council estimates of the mineral requirements of the rat. *J. Nutr.* **1966**, *89*, 495–500.
